# Dynamics of viral shedding during ancestral or Omicron BA.1 SARS-CoV-2 infection and enhancement of pre-existing immunity during breakthrough infections

**DOI:** 10.1080/22221751.2022.2122578

**Published:** 2022-10-26

**Authors:** Carla Saade, Karen Brengel-Pesce, Alexandre Gaymard, Mary-Anne Trabaud, Gregory Destras, Guy Oriol, Valérie Cheynet, Marion Debombourg, Bouchra Mokdad, Geneviève Billaud, Antoine Oblette, Hervé Créhalet, Jean-Marc Giannoli, Christine Garrigou, Laurence Generenaz, Christelle Compagnon, André Boibieux, Bruno Lina, Florence Morfin, Bruno Pozzetto, Laurence Josset, Sophie Touillet-Assant, Antonin Bal

**Affiliations:** a CIRI, Centre International de Recherche en Infectiologie, Team VirPath, Univ Lyon, Inserm, U1111, Université Claude Bernard Lyon 1, CNRS, Lyon, France; b Joint Research Unit Civils Hospices of Lyon-bioMérieux, Civils Hospices of Lyon, Lyon Sud Hospital, Pierre-Bénite, France; c Laboratoire de Virologie, Institut des Agents Infectieux, Laboratoire associé au Centre National de Référence des virus des infections respiratoires, Hospices Civils de Lyon, Lyon, France; d GenEPII sequencing platform, Institut des Agents Infectieux, Hospices Civils de Lyon, Lyon, France; e Mirialis - Biogroup Auvergne Rhône alpes, Cluses, France; f Dyomédéa-BIOGROUP – Plateau technique de la Sauvegarde, Lyon, France; g Infectious and Tropical Diseases Unit, Hospices Civil de Lyon, Lyon, France; h Team GIMAP, CIRI—Centre International de Recherche en Infectiologie, Université Jean Monnet de Saint-Etienne, Université Claude Bernard Lyon 1, Inserm, U1111, CNRS, Saint-Etienne, France; i Laboratoire des Agents Infectieux, Centre Hospitalier Universitaire de Saint-Étienne, Saint-Etienne, France

**Keywords:** SARS-CoV-2, viral shedding, Omicron, viral culture, infectiousness, vaccine-immunity

## Abstract

Omicron variant is circulating in the presence of a globally acquired immunity unlike the ancestral SARS-CoV-2 isolate. Herein, we investigated the normalized viral load dynamics and viral culture status in 44 fully vaccinated healthcare workers (HCWs) infected with the Omicron BA.1 variant. Viral load dynamics of 38 unvaccinated HCWs infected with the 20A variant during the first pandemic wave was also studied. We then explored the impact of Omicron infection on pre-existing immunity assessing anti-RBD IgG levels, neutralizing antibody titres against 19A, Delta and Omicron isolates, as well as IFN-γ release following cell stimulation with SARS-CoV-2 peptides. We reported that two weeks after diagnosis a greater proportion of HCWs infected with 20A (78.9%, 15/19) than with Omicron BA.1 (44.7%, 17/38; p = 0.02) were still positive by RT-qPCR. We found that Omicron breakthrough infections led to an overall enhancement of vaccine-induced humoral and cellular immunity as soon as a median [interquartile range] of 8 [7–9] days post symptom onset. Among samples with similar high viral loads, non-culturable samples exhibited higher neutralizing antibody titres and anti-RBD IgG levels than culturable samples. Additionally, Omicron infection led to an enhancement of antibodies neutralization capacity against other SARS-CoV-2 isolates. Taken together, the results suggest that Omicron BA.1 vaccine breakthrough infection is associated with a faster viral clearance than that of the ancestral SARS-CoV-2, in addition this new variant leads to a rapid enhancement of the humoral response against multiple SARS-CoV-2 variants, and of the cellular response.

## Introduction

The first wave of the Coronavirus Disease-19 (COVID-19) pandemic was caused by the Severe Acute Respiratory Syndrome Coronavirus-2 (SARS-CoV-2) clade 20A that had acquired the Spike (S) protein D614G mutation associated with increased transmissibility in comparison to the initial SARS-CoV-2 isolate 19A [1]. Since then, five variants of concern (VOC) have emerged, the latest being the Omicron variant, first detected in late 2021 in South Africa and largely predominant worldwide currently [2]. This SARS-CoV-2 VOC belongs to the B.1.1.529 lineage, and has 37 mutations in the S protein alone, including 15 mutations in the Receptor Binding Site (RBD), and is characterized by further increased transmissibility and a considerable escape to neutralizing antibodies [3–6].

Studies focusing on the duration of SARS-CoV-2 viral shedding have found that SARS-CoV-2 RNA can be detected using RT-PCR during several weeks after symptom onset [7–9]. It was also found that the presence of infectious virus was most frequently detected within the first 8–10 days post-symptom onset (DPSO) [7–10]. Studies addressing SARS-CoV-2 infectiousness and viral load shedding were mainly conducted prior to the emergence of the Omicron variant and these findings should not be extrapolated to this variant owing to the large number of genetic changes that it exhibits compared to the ancestral SARS-CoV-2. Furthermore, studies were also performed prior to the widespread COVID-19 vaccination campaign, which has contributed to the establishment of a global acquired immunity against SARS-CoV-2. Early studies investigating Omicron variant viral shedding have found that this variant was cleared faster than the Delta variant [11] while others have found no difference in time to negative PCR post-symptom onset between Delta and Omicron variant infections [12]. Preliminary report evaluating Omicron infectiousness looked at specific time period, such as five DPSO [13]. Given the current pandemic context, it is essential to update isolation guidelines, which are still largely based on studies performed on previous SARS-CoV-2 variants that are no longer circulating.

We aimed herein to describe viral load dynamics and infectiousness in unvaccinated healthcare workers (HCWs) infected by the ancestral SARS-CoV-2 and vaccinated HCWs infected by the Omicron VOC. Additionally, we investigated the impact of Omicron vaccine breakthrough infection (VBI) on humoral and cellular immunity dynamics.

## Methods

### Study population and design

**Figure 1.**
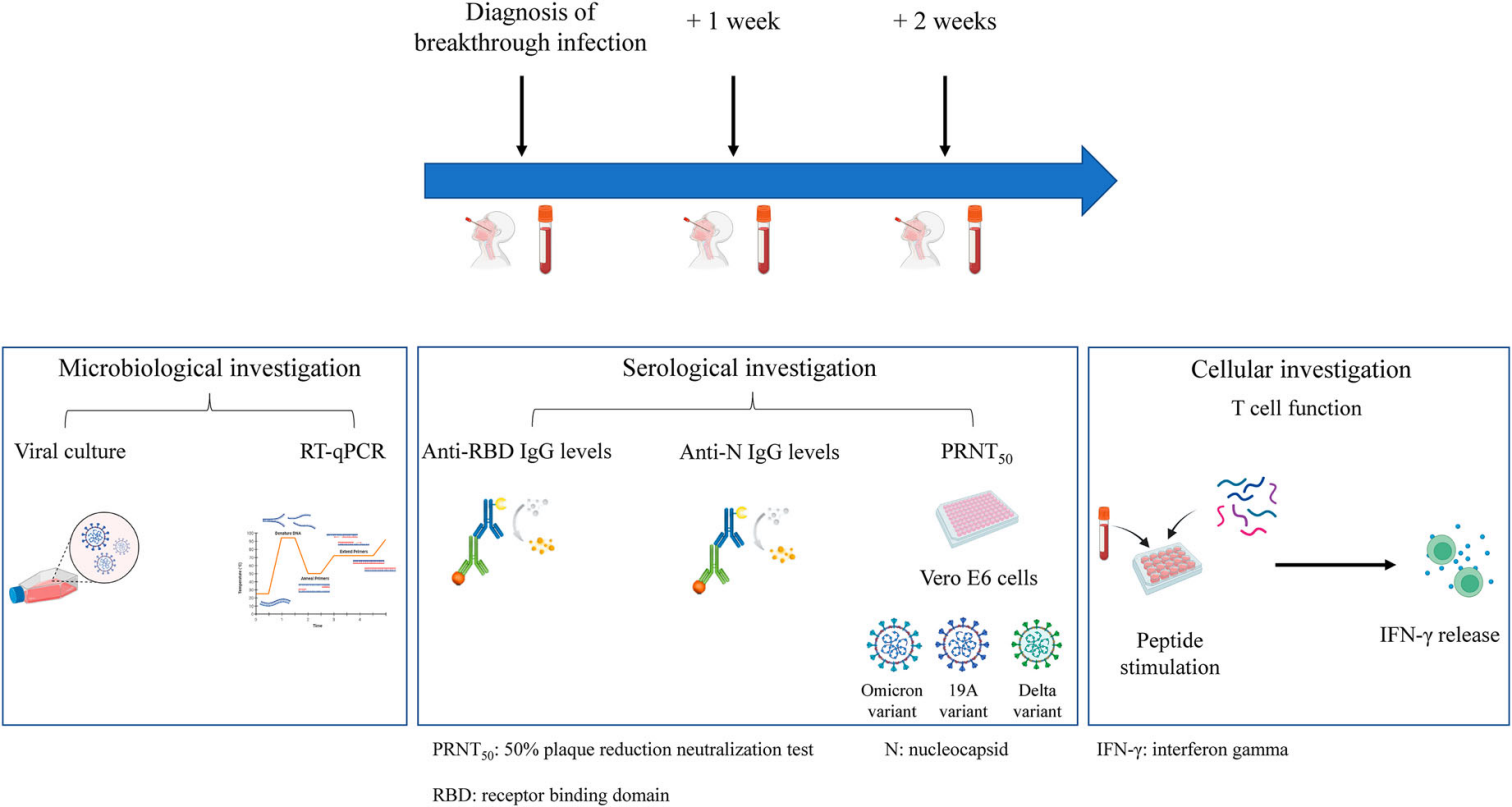
Study design for Omicron-infected healthcare workers (HCWs). A total of 44 HCWs were included. Blood samples were collected for 32 HCWs who completed all 3 visits; 12 HCWs missed at least one of the follow-up visits, nasopharyngeal swabs only were collected.

The clinical study presented herein was conducted at the National Reference Centre for Respiratory Viruses (NRC; Hospices Civil de Lyon; France). We included HCWs between November 2021 and February 2022 with Omicron BA.1 breakthrough infection having a pre-existing immunity conferred by different vaccination schemes (detailed in the supplementary material) [14]. HCWs included in this study were all fully vaccinated. Full vaccination was defined as at least 14 days after the second dose for COVID-19-naïve individuals or, at least 14 days after the first dose for convalescent individuals. They were sampled over the course of 2 weeks following symptom onset (Figure 1). Nasopharyngeal swabs (NPS) and blood samples were collected at three different time points: at diagnosis (median [interquartile range, IQR], 1 [1–3] DPSO), 1 week after diagnosis (8 [7–9] DPSO), and 2 weeks after diagnosis (15 [15–16] DPSO).

SARS-CoV-2 viral load dynamics was also described in HCWs infected in April–May 2020 [7]. These HCWs were followed longitudinally, with NPS samples collected at diagnosis (median [IQR] 2 [1–4] DPSO), 1 week after diagnosis (9 [8–10] DPSO), and 2 weeks after diagnosis (16 [15–18] DPSO).

### Microbiological investigations

The normalized viral load was determined for each sample and normalized per 1 million cells in the NPS by RT-qPCR using the SARS-CoV-2 R-gene kit (bioMérieux, Marcy l’Etoile, France). Briefly, nucleic acid extraction was performed from 0.2 mL of NPS using NUCLISENS easyMAG and amplification was performed using Bio-rad CFX96 instrument. Quantitative viral load was determined using four internally developed quantification standards (QS) targeting the SARS-CoV-2 N gene: QS1 to QS4 respectively at 1 × 10^5^, 1 × 10^4^, 1 × 10^3^, 1 × 10^2^ copies/µL of a SARS-CoV-2 DNA standard. These QS were controlled and quantified using the Nanodrop spectrophotometer (Thermo Fisher Scientific, MA, USA) and Applied Biosystems QuantStudio 3D Digital PCR. In parallel, NPS were tested using the CELL Control R-GENE kit (amplification of the HPRT1 housekeeping gene, bioMérieux) that contains two quantification standards QS1 and QS2, at 10^4^ copies/µL (50,000 cells/PCR in our conditions) and 10^3^ copies/µL (5,000 cells/PCR) of DNA standard, respectively, to normalize the viral load according to the sampling quality.

$$Normalized\;viral\;load[Log_{10}cp/10^{6}\;cells]=Log10\;\left[\frac{number\;of\;SARS\text{-}CoV\text{-}2\;copies}{number\;of\;cells}\times 10^{6}\;cells\right]$$

Viral culture was also conducted for each collected sample as previously described [7]. Viral culture was performed following interim biosafety guidelines established by the World Health Organization (WHO) [15] from NPS in universal transport medium – room temperature (UTM-RT). RT-PCR-positive NPS were stored at −80°C prior to viral culture. These samples were inoculated on confluent Vero-E6 cells (ATCC CCL-81) maintained in Eagle’s Minimum Essential Media (EMEM) supplemented with 2% penicillin–streptomycin, 1% L-glutamine, and 2% inactivated foetal bovine serum. Plates were incubated at 33°C in an atmosphere containing 5% CO_2_ for 96 h. The cytopathic effects (CPE) were monitored daily; samples were harvested when positive, while negative samples at 96 h underwent subculture on new plates. Culture supernatants were sampled at 2 h post-inoculation, at 96 h, and after an additional 96 h of subculture. RNA from supernatants was extracted using the automated MGISP960 workstation using MGI Easy Magnetic Beads Virus DNA/RNA Extraction Kit (MGI Tech, Marupe, Latvia), and SARS-CoV-2 detection was performed using TaqPath COVID-19 CE-IVD RT-PCR kit on a QuantStudio 5 System (Thermo Fisher Scientific).

SARS-CoV-2 whole-genome sequencing was performed at the national reference centre for respiratory viruses using the artic V3 [16] or artic V4.1 protocol [17] for ancestral and Omicron samples, respectively.

### Humoral immune response

For a subset of HCWs infected with Omicron (*n* = 32 HCWs, *n* = 96 samples), anti-RBD IgG levels were determined at each visit using the Siemens Healthineers (Erlangen, Germany) Atellica® IM SARS-CoV-2 IgG (sCOVG), according to the protocol recommended by the manufacturer and expressed in binding antibody units (BAU)/mL.

Anti-N IgG levels were also determined for these samples using the Abbott (Sligo, Ireland) Architect® SARS-CoV-2 IgG, according to the protocol recommended by the manufacturer.

A live virus neutralization test measuring neutralizing antibodies titres against 19A, Delta and Omicron (BA.1) isolates, was also performed on the same subset of HCWs infected with Omicron (*n* = 32 HCWs, *n* = 96 samples). GISAID accession numbers are EPI_ISL_1707038, EPI_ISL_1904989 and EPI_ISL_7608613 for the 19A, Delta and Omicron isolates, respectively. These experiments were performed in a biosafety level 3 laboratory as previously described [18,19]. Briefly, viral variants used for these experiments were cultured on Vero-E6 cells. Each serum tested was diluted 1:10 and serial twofold dilutions were mixed with an equal volume (100 µL) of virus. After gentle shaking and an incubation for 30 min at room temperature, 150 µL of each mixture was transferred to 96-well microplates covered with Vero-E6 cells. Then, the plates were incubated at 37°C in an atmosphere containing 5% CO_2_. Measurements were obtained microscopically 5–6 days later when the cytopathic effect of the virus control reached ∼100 TCID_50_/150 µL (50% tissue culture infectious dose). The serum was considered to have protected the cells if >50% of the cell layer was preserved. The neutralizing titre was expressed as the inverse of the higher serum dilution that protected the cells.

### Cellular immune response

In addition, the cellular response was investigated using an interferon-gamma (IFN-γ) release assay (IGRA) as previously described [20] using the VIDAS® COVID stimulation and VIDAS® 9IFN Research Use Only (RUO) kits (bioMérieux). These experiments were performed on a subset of samples from HCWs infected with Omicron (*n* = 24 HCWs, *n* = 72 samples). In brief, whole blood was stimulated with a restricted pool of peptides (RPP) specific to SARS-CoV-2 structural proteins. The supernatant was then collected 22 h post-stimulation and IFN-γ release was quantified using an automated VIDAS® ELISA test to determine IFN-γ levels for each sample. Manufacturer’s instructions were followed for the determination of the positivity threshold.

### Statistical analysis

The viral load kinetics of the 20A and the Omicron variant were represented using a multiple linear regression model followed by a Fisher test and as needed by a *t*-test. Samples were also stratified according to viral culture status for both variants; the normalized viral loads between the culturable and non-culturable samples were compared using the Mann–Whitney *U*-test. In addition, anti-RBD IgG levels, neutralizing antibody titres, and IFN-γ concentrations of the three time points were compared using the Friedman test followed by the Dunn’s multiple comparison test. Analyses were conducted using GraphPad Prism® software (version 8; GraphPad software, La Jolla, CA, USA) and R software, version 3.6.1 (R Foundation for Statistical Computing, Vienna, Austria).

### Study approval

The study presented herein is registered on ClinicalTrial.gov (NCT04341142) [21]. Written informed consent was obtained from all participants and approval was obtained from the regional review board in April 2020 (*Comité de Protection des Personnes Sud Méditerranée I*, Marseille, France; ID-RCB 2020-A00932-37).

## Results

### Clinical characteristics of HCWs with Omicron breakthrough infection

A total of 44 fully vaccinated HCWs with a positive SARS-CoV-2 RT-qPCR were included between November 2021 and February 2022. Later SARS-CoV-2 whole-genome sequencing confirmed that infections were caused by the lineage BA.1 of the Omicron variant. All HCWs included presented symptoms of COVID-19 but none were hospitalized.

The majority of HCWs experienced a sore throat (65.9%, 29/44). Less than 50% of HCWs experienced fever (45.4%, 20/44), muscular pain (15.9%; 7/44), asthenia (15.9%, 7/44), cough (40.9%, 18/44), shortness of breath (6.8%, 3/44), headache (38.6%, 17/44), and ageusia (4.5%, 2/44) (Table 1).

**Table 1.** Demographic and clinical characteristics of healthcare workers (HCWs) infected by Omicron.

	Omicron (*n* = 44)
Sex, female, *n* (%)	32 (72.7)
Age, years, median [IQR]	32.5 [25.8–45]
Body mass index, median [IQR]	22 [20.8–27.1]^a^
Presence of comorbidity (ies), *n* (%)	8/44 (18.18)
Neurological disorders, *n* (%)	0 (0)
Hypertension, *n* (%)	1 (11.1)
Heart failure, *n* (%)	1 (11.1)
Diabetes, *n* (%)	0 (0)
Immune deficiency, *n* (%)	1 (11.1)
Liver disease, *n* (%)	0 (0)
Kidney disease, *n* (%)	1 (11.1)
Cancer, *n* (%)	1 (11.1)
Hypothyroid, *n* (%)	1 (11.1)
Rheumatic disease, *n* (%)	1 (11.1)
Chronic respiratory disease, *n* (%)	1 (11.1)
Post-symptom delay, day, median [IQR]	1 [1-3]
Symptoms^b^	
Fever, *n* (%)	20 (45.4)
Sore throat, *n* (%)	29 (65.9)
Muscular pain, *n* (%)	7 (15.9)
Asthenia, *n* (%)	7 (15.9)
Rhinorrhoea, *n* (%)	18 (40.9)
Cough, *n* (%)	18 (40.9)
Shortness of breath, *n* (%)	3 (6.8)
Headache, *n* (%)	17 (38.6)
Anosmia, *n* (%)	0 (0)
Ageusia, *n* (%)	2 (4.5)
Ophthalmic pain, *n* (%)	1 (2.2)

IQR: inter-quartile range.

^a^ 41 subjects instead of 44 (3 missing values).

^b^ Symptoms were recorded at inclusion, i.e. at diagnosis.

### Viral load dynamics and culturability

One week after diagnosis of SARS-CoV-2 infection, more than 90% of HCWs had a positive RT-qPCR (90.4% vs. 90.9% for the Omicron and 20A groups, respectively). Two weeks after diagnosis a greater proportion of HCWs infected with 20A (78.9%, 15/19) than with Omicron (44.7%, 17/38; *p* = 0.02) were still positive by RT-qPCR.

**Figure 2.**
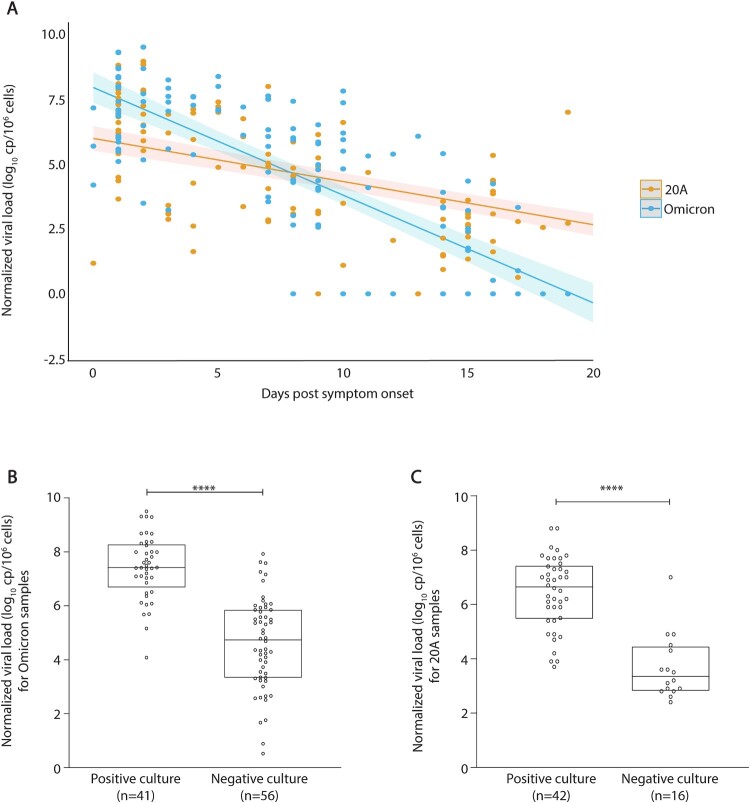
Viral kinetics and viral load according to viral culture status for 20A- and Omicron- infected healthcare workers. Kinetics of viral infection caused by the SARS-CoV-2 20A variant or the Omicron variant (A). The mean number of HCWs sampled per day was three for 20A-infected HCWs and six for Omicron-infected HCWs. Curves representing the kinetic of the viral infection by the first wave variant 20A (in orange), or by the Omicron variant (in blue); within the first 20 days following symptom onset. The light-coloured area around the curves represents the confidence interval of each curve. Box plots represent the median [interquartile range, IQR] of the normalized SARS-CoV-2 viral loads (log_10_ cp/10^6^ cells) stratified according to viral culture status for the Omicron variant (B) and the 20A variant (C) among SARS-CoV-2 positive samples. The median [IQR] of the normalized viral load was compared between culturable and non-culturable samples for Omicron-infected HCWs (7.4 [6.6–8.2] log_10_ cp/10^6^ cells vs. 4.7 [3.3–5.4] log_10_ cp/10^6^ cells, *p* < 0.0001; B) as well as for 20A-infected HCWs (6.6 [5.4–7.4] log_10_ cp/10^6^ cells vs. 3.5 [2.9–4.6] log_10_ cp/10^6^ cells, *p* < 0.0001; C). Comparisons were made using the Mann–Whitney *U*-test. ****p < 0.0001.

A decrease of 0.42 log_10_ cp/10^6^ cells in viral load was noted in HCWs infected with Omicron for each additional day after symptom onset while a decrease of 0.17 log_10_ cells/10^6^ cells was noted for HCWs infected with 20A for each additional day after symptom onset (*p* < 0.001; Figure 2(A)).

Regarding the frequency of virus isolation in samples from HCWs infected with Omicron we reported an isolation frequency of 67.4% (29/43) at diagnosis, 28.5% (12/42) 1 week after diagnosis and 0% (0/38) 2 weeks after diagnosis. For reference, the frequency of virus isolation in samples from HCWs infected with 20A was 89.4% (34/38) at diagnosis, 60% (6/10) 1 week after diagnosis and 20% (2/10) 2 weeks after diagnosis.

Of note, higher median [IQR] normalized viral loads were found in culturable samples than in non-culturable samples for both HCWs infected with Omicron (Figure 2(B), Supplementary Figure S1(A,C,E)) and 20A (Figure 2(C), Supplementary Figure S1(B,D,F)).

### Enhancement of pre-existing immunity following Omicron infection

While HCWs infected during the first wave had no evidence of pre-existing SARS-CoV-2 immunity, all Omicron infected HCWs received a full vaccination scheme before being infected by the new variant. Thus, the impact of Omicron breakthrough infection on pre-existing humoral and cellular immunity was assessed.

**Figure 3.**
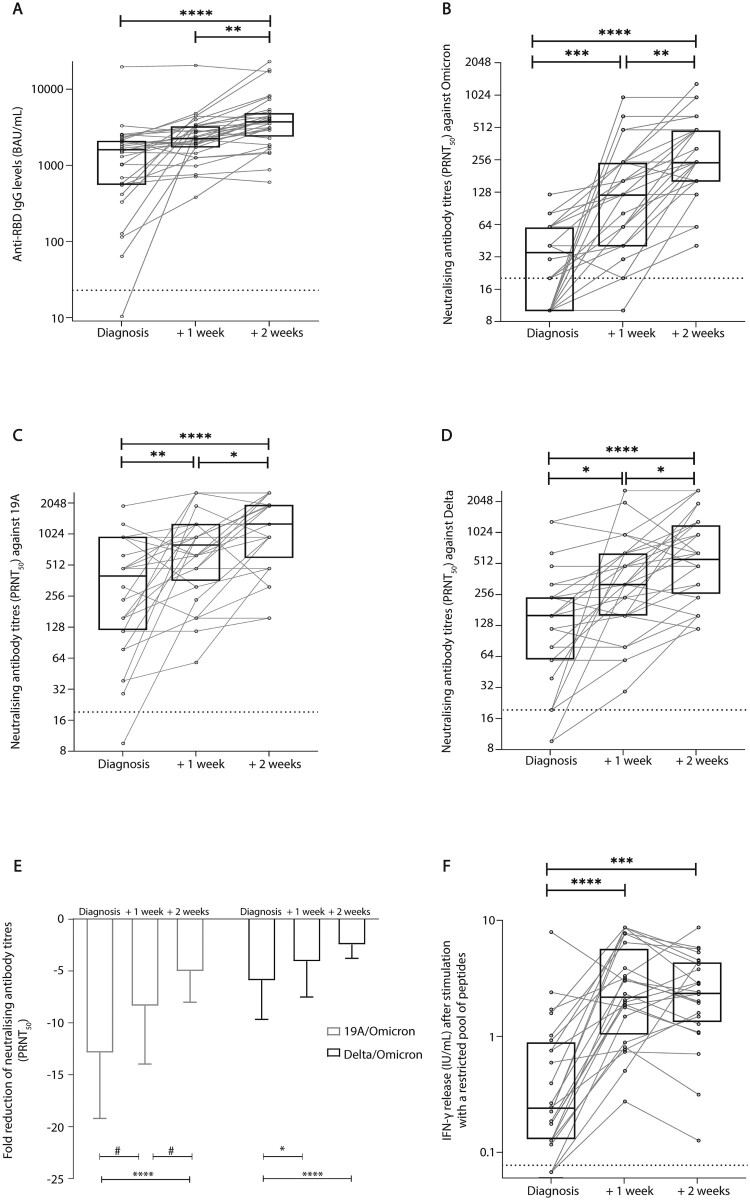
Immunological parameters assessed up to 2 weeks post Omicron infection. A chemiluminescence immunoassay (CLIA) was performed in order to determine anti-RBD IgG levels (A), expressed in binding antibody units/mL (*n* = 96). The dotted line represents the threshold of positivity (≥21.8 BAU/mL). Neutralizing antibody titres (PRNT_50_) against the Omicron (B), 19A (C) and Delta (D) isolates (*n* = 96). The dotted line represents the threshold of positivity (a titre ≥ 20). Fold reduction were calculated for Omicron in comparison to 19A and Delta. Bar plot represents the mean fold change with standard deviation at diagnosis (*n* = 21), 1 week after (*n* = 32) and 2 weeks after (*n* = 32) (E). An Interferon Gamma Release Assay was performed to assess T cell function following stimulation with a restricted pool of peptides (RPP) derived from SARS-CoV-2 structural proteins (*n* = 72) (F). The dotted line represents the threshold of positivity (≥0.08 IU/mL). For all three experiments (A–D, F), box plots represent the median [IQR] for each parameter and before-and-after scatter plots represent the evolution of the parameter for each patient individually. Samples were obtained at diagnosis, 1 week after diagnosis, and 2 weeks after diagnosis. Comparisons were made using the Friedman test followed by Dunn’s multiple comparison test. *p = 0.05; **p < 0.01; ***p < 0.001; ****p < 0.0001; ^#^*p* < 0.1.

For HCWs infected with Omicron and who completed all three visits (*n* = 32 HCWs, *n* = 96 samples), the median [IQR] anti-RBD IgG levels was 1603 [554.1–2100] BAU/mL at diagnosis, 2245 [1719–3250] BAU/mL 1 week after diagnosis, and 3721 [2389–4841] BAU/mL 2 weeks after diagnosis (Figure 3(A)). Moreover, anti-N IgG levels were also determined (Supplementary Figure S2).

The same 96 samples were tested by live virus neutralization test. There was an increase in the median [IQR] neutralizing antibody titres against Omicron over time; 35 [10–60] at diagnosis and 240 [160–480] 2 weeks after (*p* < 0.0001; Figure 3(B)). It is worth noting that a significant increase in the median neutralizing antibody titres, between diagnosis and week 2, was also observed against the 19A and the Delta variant (Figure 3(C,D) respectively). The respective fold change in neutralizing antibody titres between 19A and Omicron, and Delta and Omicron was calculated. At diagnosis, 11/32 (34.3%) samples that did not have any detectable neutralizing antibodies were excluded from the analysis at that time point. A 12.8-fold and 5.8-fold decrease were recorded against Omicron in comparison to 19A and Delta, respectively, at diagnosis. Conversely, 2 weeks after diagnosis, the fold change of neutralizing antibody titres was lower, with a 5.0-fold and a 2.4-fold decrease in comparison to 19A and Delta, respectively (Figure 3(E)).

Among those tested, 87.5% (21/24) of samples were positive for IFN-γ release at diagnosis, 24/24 (100%) 1 week after diagnosis, and 24/24 (100%) 2 weeks after diagnosis for HCWs infected with Omicron. The median [IQR] concentration of IFN-γ release at 1 week after diagnosis (2.1 [1.0–5.6] IU/mL) was significantly higher than that found at diagnosis (0.2 [0.1–0.8] IU/mL, *p* < 0.0001; Figure 3(F)).

### Association of cellular and humoral immunity with viral culturability

IFN-γ release levels were stratified according to virus isolation status for HCWs infected with Omicron (culturable vs. non-culturable samples). No significant difference in the median IFN-γ release was observed between HCWs with culturable and non-culturable viruses in respiratory samples at any time point (*p* = 0.08 at diagnosis, Supplementary Figure S3(A), *p* = 0.71 1 week after diagnosis, Supplementary Figure S3(B)).

**Figure 4.**
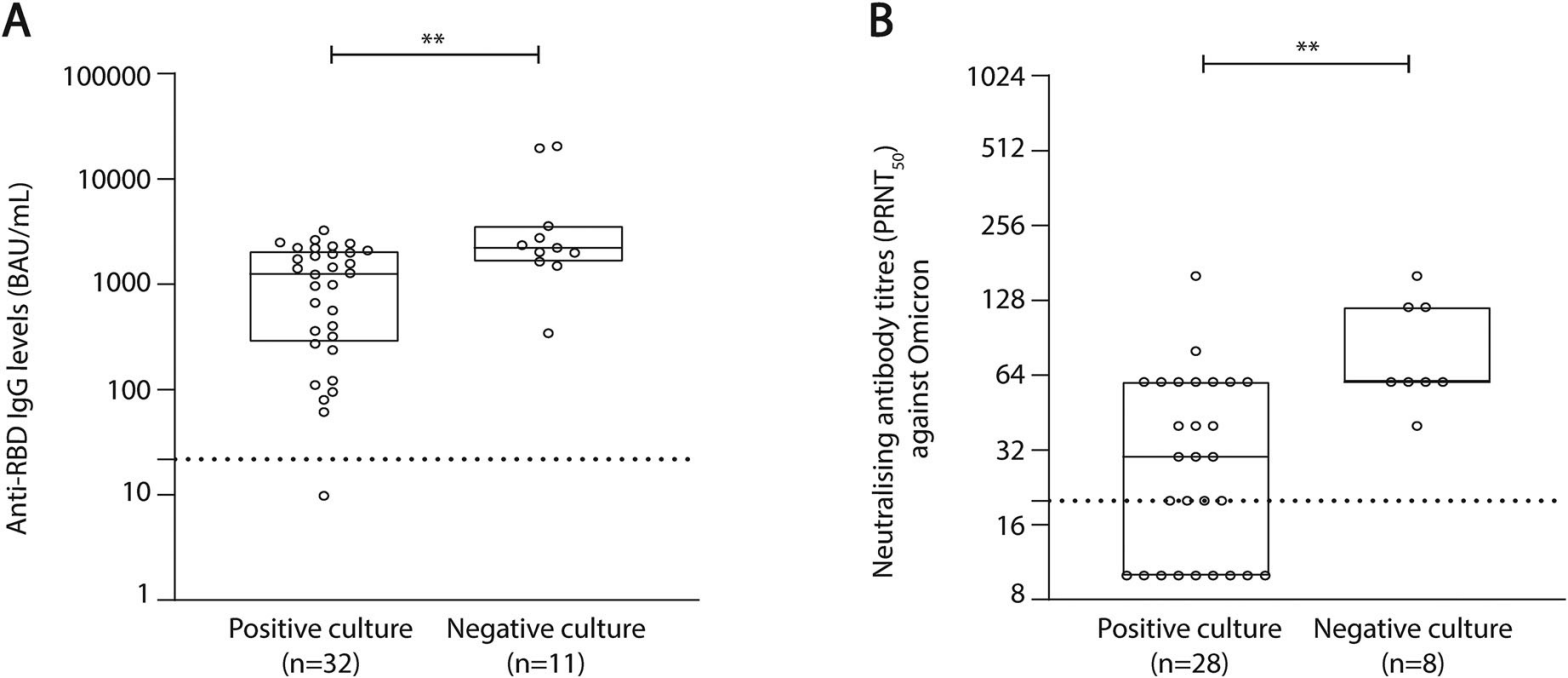
Anti-RBD IgG levels and neutralizing antibody titres stratified according to isolation status among HCWs infected with Omicron. Samples having a similar high viral load (≥6 log_10_ cp/10^6^ cells) were selected. A chemiluminescence immunoassay (CLIA) was performed in order to determine anti-RBD IgG levels, expressed here in binding antibody unit/mL (BAU/mL) and stratified according to culture status (A). The dotted line represents the threshold of positivity (≥21.8 BAU/mL). A 50% plaque reduction neutralization test (PRNT_50_) was conducted to determine the neutralizing antibody titre of each sample against the live Omicron variant and stratified according to culture status (B). The dotted line represents the threshold of positivity (a titre ≥ 20). For both experiments, box plots represent the median [IQR] for each parameter and before-and-after scatter plots represent the evolution of the parameter for each patient individually. Comparisons were made using the Mann–Whitney *U*-test. **p < 0.01.

In order to evaluate the impact of anti-RBD IgG titres and neutralizing antibodies on viral culturability level, we compared these parameters in NPS with similar high viral loads (≥6 log_10_ cp/10^6^ cells). Significantly higher median [IQR] anti-RBD IgG levels were detected for non-culturable samples in comparison to culturable samples, 2212 [1641–3558] BAU/mL vs. 1254 [284.4–2067] BAU/mL respectively (*p* = 0.004, Figure 4(A)). Additionally, neutralizing antibody titres were 2-fold higher in non-culturable samples in comparison to culturable samples, 60 [60–120] vs. 30 [10–60], respectively (*p* = 0.0017, Figure 4(B)).

## Discussion

Isolation guidelines are still largely based on SARS-CoV-2 variants that are no longer circulating. It is thus necessary to study viral and immune kinetics given the current pandemic context in order to update isolation guidelines, especially for HCWs. In the present study, we selected a population of HCWs totally naïve for SARS-CoV-2 infected during the first wave versus the same type of population with previous immunization against SARS-CoV-2 through full vaccination infected with Omicron BA.1.

To our knowledge, this is the first study describing viral load dynamics of Omicron BA.1 infected HCWs and HCWs infected during the first SARS-CoV-2 wave. We found a faster viral clearance in HCWs infected with Omicron BA.1 than with 20A. Hay et al. reported a lower viral peak for Omicron in comparison to Delta as well as a faster clearance [11]. Kissler et al. reported no significant difference in viral infection dynamics between infections caused by Alpha, Delta and non-VOC variants. As well, Kissler et al. reported faster viral clearance for breakthrough infections in comparison to unvaccinated individuals [22]. In addition, a study reported by Jung et al. found that Delta viral load in fully vaccinated individuals was similar to that in unvaccinated individuals, however, full vaccination led to a faster decrease in viral viability [23]. Overall, these last two studies showed that vaccination led to a faster decrease in viral load. Thus it is still not clear whether the faster decrease in Omicron viral load, reported herein, is due to intrinsic characteristics acquired by Omicron or due to the widespread vaccination, or both. Although it is not possible to determine the exact reason for the observed differences, these descriptions provide information as to the evolution of the pandemic regarding viral infection dynamics between the ancestral variant and the latest VOC that has emerged. Especially in the context of conflicting results, such as those reported by Boucau et al. indicating similar median duration of viral shedding between Delta- and Omicron-infected individuals. Furthermore, Boucau et al. reported no large differences in the median duration of viral shedding between unvaccinated and vaccinated individuals [12].

Although the viral load is often used as a proxy of infectiousness, the presence of infectious virus assessed by viral culture is a much better predictor of this parameter [13]. Herein, virus isolation frequency was lower for Omicron BA.1 in comparison to 20A, at all 3 visits, suggesting a more efficient control of virus replication. In the study reported by Puhach et al., the infectious viral load was compared between vaccinated and unvaccinated individuals following Omicron infection; these authors found a significant reduction in infectious viral load in boosted vaccinated subjects but not in fully vaccinated or in unvaccinated subjects [13]. This would suggest that additional vaccine doses would help decrease viral load even further and reduce viral infectiousness.

Another objective of this study was to investigate the impact of Omicron infection on pre-existing immunity, acquired by either a previous infection followed by vaccination or vaccination alone.

In the present study, Omicron infection, in all vaccinated HCWs, led to an increase in neutralizing antibody titres against three SARS-CoV-2 isolates; 19A, Delta and Omicron, indicating an increase in broad neutralizing capacity of antibodies. Additionally, herein we report that the fold change of neutralizing antibody titres against Omicron in comparison to the 19A and Delta isolates were of smaller weight 2 weeks after diagnosis than at diagnosis. This suggests that Omicron infections promote the production of antibodies that can neutralize multiple SARS-CoV-2 isolates with a relatively equal neutralization capacity. The gap in neutralization capacity between different SARS-CoV-2 variants is reduced following an Omicron infection of a vaccinated individual. Similar results have been reported by Khan et al., who showed that Omicron infection in previously vaccinated individuals led to higher neutralization levels of Omicron as well as Delta and Beta in comparison to non-vaccinated individuals [24]. These findings would indicate that Omicron infection and vaccination could decrease the incidence of future infections caused by other SARS-CoV-2 variants.

When stratifying Omicron samples by culture status we found no significant difference in the median IFN-γ concentration between culturable and non-culturable samples, indicating that the T cell response was not significantly associated with viral infectiousness. When a subset of samples from HCWs infected with Omicron and having a similar high viral load ≥ than 6 log_10_ cp/10^6^ cells were selected, higher anti-RBD IgG levels and neutralizing antibody titres were found for non-culturable samples in comparison to culturable samples. This suggests that high levels of anti-RBD antibodies and neutralizing antibodies could be involved in the reduction of viral infectiousness.

The present study has some limitations. First, the comparison between viral variants regarding viral isolation should be made with reservations as the sensitivity of viral culture could vary according to the variant. Second, investigation of immunological parameters such as the presence of neutralizing antibodies was made on a small-sized and non-paired effective of 20A-infected HCWs; we therefore could not compare these results to those of Omicron infections. Additionally, only a small number of NPS samples of 20A-infected HCWs was assessed for culturability at 1 and 2 weeks post diagnosis. Third, the IGRA test was not normalized to the number of cells present in the sample and quantitative results should be interpreted with caution. Fourth, Omicron-infected HCWs were sampled only three times over the course of the first 2 weeks, which might have been too infrequent to observe rapid kinetic changes of viral load and antibody titres. Finally, since vaccination is mandatory for HCWs, we did not include unvaccinated Omicron patients, which hampered the assessment of the impact of vaccination on Omicron infection. As well, included HCWs infected during the first wave were all naïve to SARS-CoV-2 and thus we could not assess the impact of vaccination of 20A-viral kinetics.

In conclusion, the results of the present study suggest distinct viral load dynamic patterns during infections with the ancestral SARS-CoV-2 variant and the latest VOC that has emerged. These results could contribute to optimize current isolation guidelines that are mainly based on studies conducted during the first wave. Whether these differences are related to vaccine-acquired immunity or to intrinsic properties of the Omicron variant needs further investigations. Additionally, results presented herein suggest that Omicron infection leads to a rapid enhancement of the humoral response, specifically neutralization capacity, against Omicron as well as other SARS-CoV-2 variants.

## Author contributions

S.T.-A., K.B.-P., A.G., F.M.S., L.J., B.L. and A.B. conceived the study, planned, designed and interpreted experiments. C.S. and A.B. wrote the initial draft. A.O., A.G., F.M., L.J., G.D., performed viral culture, RT-PCR, whole-genome sequencing and interpret analysis. Serological and neutralization assays were performed by M.-A.T., C.C., L.G., C.S., M.D., B.P., F.M.S., V.C., A.G., K.B.-P.

B.M., G.B., H.C., J.-M.G., C.G., A.B. provided clinical samples and critically reviewed patient data. G.O. and C.S. performed statistical analysis and generated figures. Covid ser Study group members significantly contributed to clinical, biological and viral data and patient’s follow up. All authors reviewed the manuscript and agreed to its submission.

## Supplementary Material

Supplemental Material

## Data Availability

SARS-CoV-2 whole genome sequencing was performed on all NPS at diagnosis. High quality sequences were submitted to GISAID and their accession numbers were included in the supplementary materials (*n* = 29 for 20A-infected HCWs and *n* = 42 for Omicron-infected HCWs). SARS-CoV-2 isolates used for the live neutralization were sequenced prior to the experiments and the sequences were submitted to GISAID.
