# Supplementary material for: Dynamics of viral shedding during ancestral or Omicron BA.1 SARS-CoV-2 infection and enhancement of pre-existing immunity during breakthrough infections
Source: Emerg Microbes Infect. 2022 Oct 26;11(1):2423–32. doi: 10.1080/22221751.2022.2122578 (PMC9621261; doi:10.1080/22221751.2022.2122578)
Supplement: Supplemental Material [file TEMI_A_2122578_SM5624.zip › Supplementary_material/Supplementary figure legends_EMI_R2_225703664.docx]

**Supplementary Figure S1: Normalized viral load (log_10_ cp/10^6^ cells) for 20A and Omicron at diagnosis, 1 week after and 2 weeks after stratified according to viral isolation status**

Box plots represent the median [IQR] of the normalized viral at diagnosis, 1 week after diagnosis, and 2 weeks after diagnosis for the first wave variant, 20A (A,C,E), and Omicron (B,D,F) among SARS-CoV-2 positive samples. Relevant statistical analysis could not be conducted due to the low sample number.

**Supplementary Figure S2: anti-N IgG levels (index) were determined for Omicron-infected HCWs**

Anti-N IgG levels were quantified at diagnosis, 1 week after diagnosis, and 2 weeks after diagnosis; using the Architect Abbot enzyme linked immunosorbent assay (ELISA). Anti-N IgG were only detected in convalescent patients. Box plots represent the median [IQR] and scatter plots represent anti-N IgG levels for each patient individually. Dotted line represents the positivity threshold (index ≥ 1.4). Comparisons were made using the Friedman test followed by Dunn’s multiple comparison test. **p<0.01; ****p<0.0001.

**Supplementary Figure S3: IFN-γ release assay stratified according to virus isolation status for Omicron-infected HCWs at diagnosis and 1 week after**

An Interferon Gamma Release Assay was performed to assess T cell function following stimulation with a restricted pool of peptides (RPP) derived from SARS-CoV-2 structural proteins. The assay was performed at diagnosis (A) and 1 week after diagnosis (B). The dotted lines represent the threshold of positivity for each test (≥ 0.08 IU/mL). Comparisons were made using the Mann-Whitney U-test.
