# Supplementary material for: Dynamics of viral shedding during ancestral or Omicron BA.1 SARS-CoV-2 infection and enhancement of pre-existing immunity during breakthrough infections
Source: Emerg Microbes Infect. 2022 Oct 26;11(1):2423–32. doi: 10.1080/22221751.2022.2122578 (PMC9621261; doi:10.1080/22221751.2022.2122578)
Supplement: Supplemental Material [file TEMI_A_2122578_SM5624.zip › Supplementary_material/Supplementary material_EMI_R1_225703664.docx]

**Supplementary material:**

**Description of vaccination scheme for HCWs infected with Omicron**

HCWs had one of the following vaccination schemes: i) naïve-HCWs who received 2 doses of the BNT162b2 mRNA vaccine (n=11), ii) naïve-HCWs who received 3 doses of the BNT162b2 mRNA vaccine (n=12), iii) naïve-HCWs with a heterologous vaccination scheme defined as 1 dose of the ChadOx-1-S-nCoV-19 vaccine followed by either 1 or 2 doses of an mRNA-based vaccine (n=13), and iv) HCWs with hybrid immunity defined as a previous infection followed by either one or two doses of vaccine depending on the date of their previous infections (n=8). Delay between last vaccine dose and infection was variable, with a median of 83 [40-172] days.

**GISAID accession numbers relative to strains detected in the nasopharyngeal swabs sampled**

| Samples from HCWs infected with 20A | Samples from HCWs infected with Omicron |
| --- | --- |
| EPI_ISL_13955207 | EPI_ISL_8121151 |
| EPI_ISL_13955229 | EPI_ISL_13805472 |
| EPI_ISL_13955208 | EPI_ISL_13805471 |
| EPI_ISL_13955209 | EPI_ISL_13805470 |
| EPI_ISL_13955230 | EPI_ISL_8121878 |
| EPI_ISL_13955231 | EPI_ISL_8121871 |
| EPI_ISL_13955210 | EPI_ISL_8298106 |
| EPI_ISL_13955232 | EPI_ISL_8298100 |
| EPI_ISL_13955211 | EPI_ISL_8298101 |
| EPI_ISL_13955233 | EPI_ISL_8298097 |
| EPI_ISL_13955212 | EPI_ISL_6963002 |
| EPI_ISL_13955213 | EPI_ISL_8673463 |
| EPI_ISL_13955214 | EPI_ISL_9426590 |
| EPI_ISL_13955215 | EPI_ISL_8673421 |
| EPI_ISL_13955216 | EPI_ISL_9426559 |
| EPI_ISL_13955217 | EPI_ISL_9426558 |
| EPI_ISL_13955218 | EPI_ISL_9156239 |
| EPI_ISL_13955219 | EPI_ISL_9156240 |
| EPI_ISL_13955220 | EPI_ISL_9156241 |
| EPI_ISL_13955221 | EPI_ISL_9156236 |
| EPI_ISL_13955222 | EPI_ISL_9156237 |
| EPI_ISL_13955223 | EPI_ISL_9156238 |
| EPI_ISL_13955224 | EPI_ISL_9156242 |
| EPI_ISL_13955225 | EPI_ISL_9156249 |
| EPI_ISL_13955226 | EPI_ISL_9322611 |
| EPI_ISL_13955205 | EPI_ISL_9322625 |
| EPI_ISL_13955227 | EPI_ISL_13805469 |
| EPI_ISL_13955206 | EPI_ISL_13805473 |
| EPI_ISL_13955228 | EPI_ISL_13805474 |
|  | EPI_ISL_13805475 |
|  | EPI_ISL_9322621 |
|  | EPI_ISL_13805476 |
|  | EPI_ISL_9603626 |
|  | EPI_ISL_9603644 |
|  | EPI_ISL_9603623 |
|  | EPI_ISL_9322629 |
|  | EPI_ISL_9603690 |
|  | EPI_ISL_9603688 |
|  | EPI_ISL_9603696 |
|  | EPI_ISL_9603824 |
|  | EPI_ISL_9603829 |
|  | EPI_ISL_9603826 |
