# Supplementary figures and images for: Dynamics of viral shedding during ancestral or Omicron BA.1 SARS-CoV-2 infection and enhancement of pre-existing immunity during breakthrough infections
Source: Emerg Microbes Infect. 2022 Oct 26;11(1):2423–32. doi: 10.1080/22221751.2022.2122578 (PMC9621261; doi:10.1080/22221751.2022.2122578)

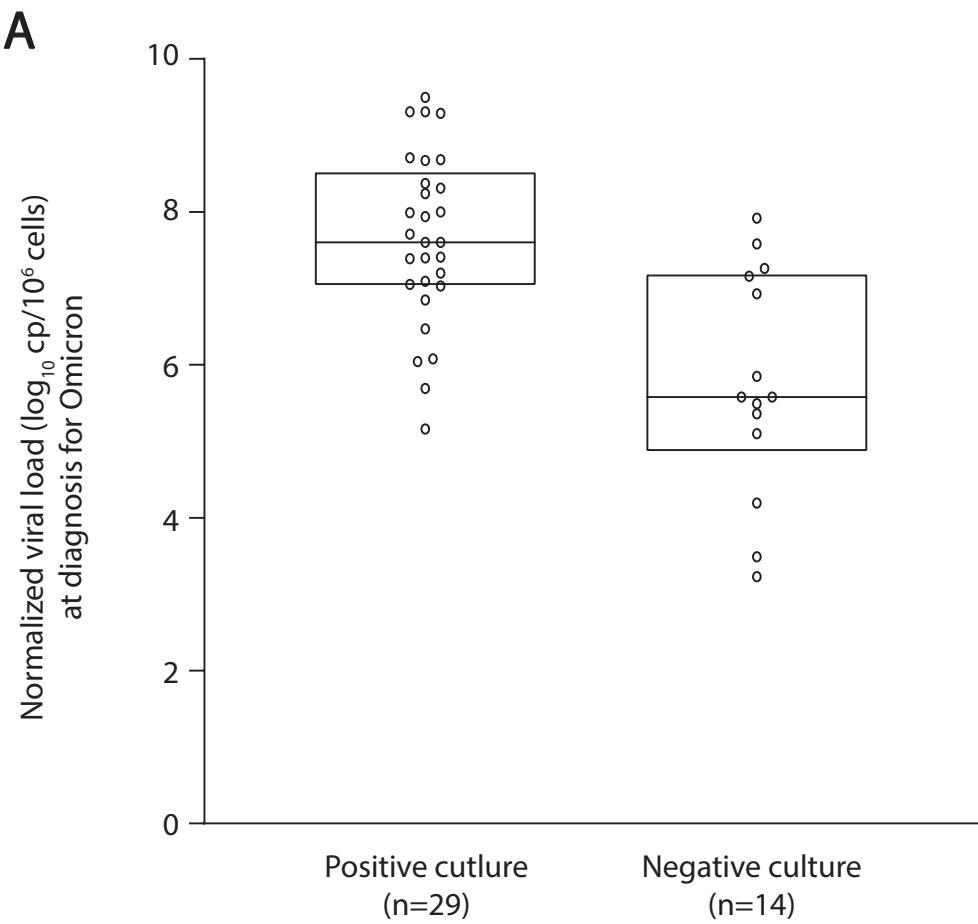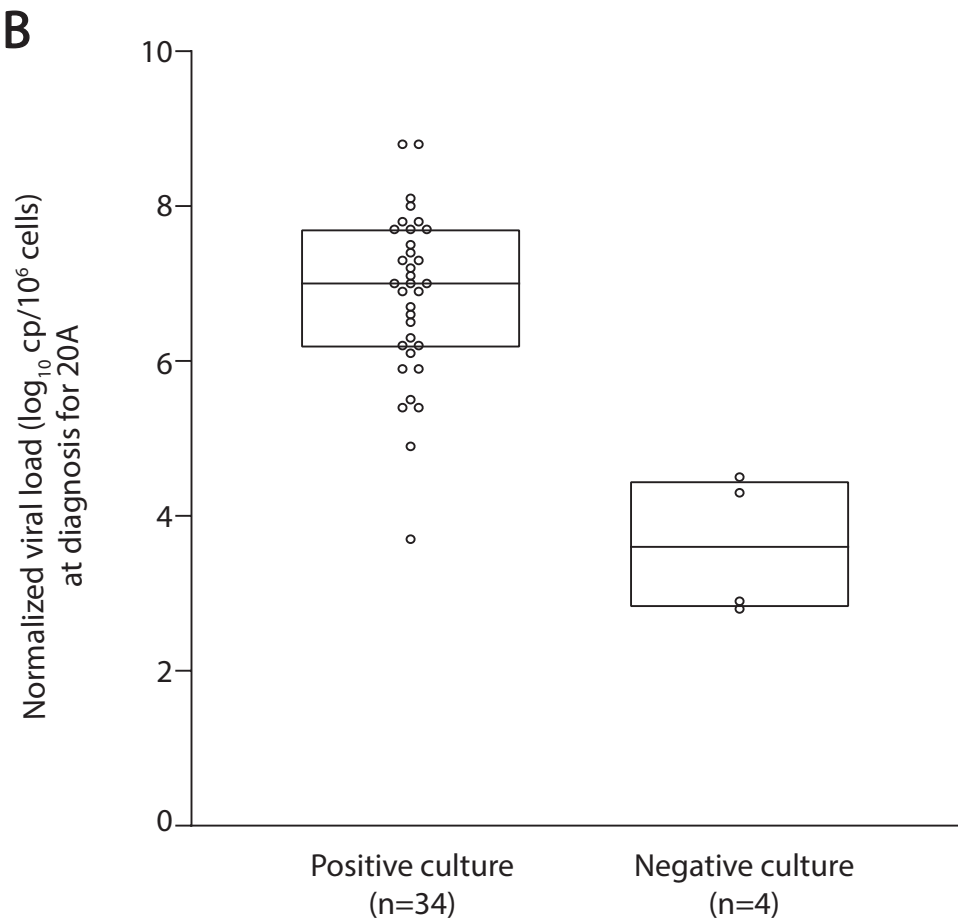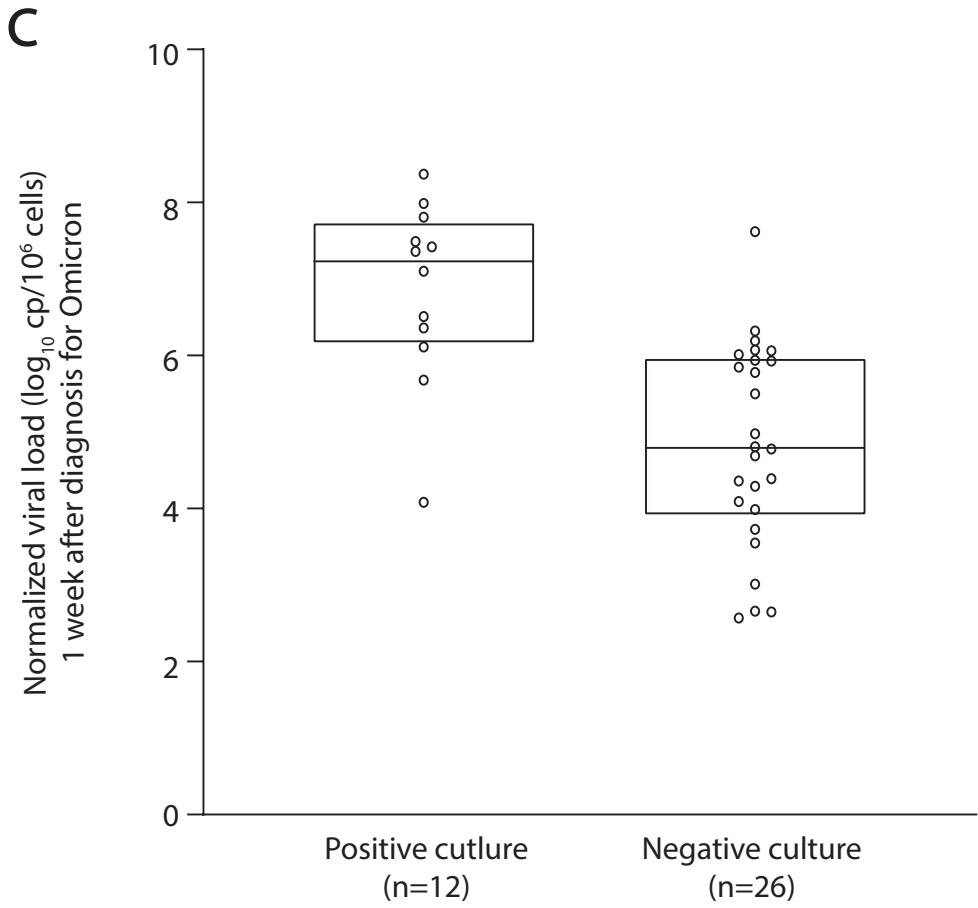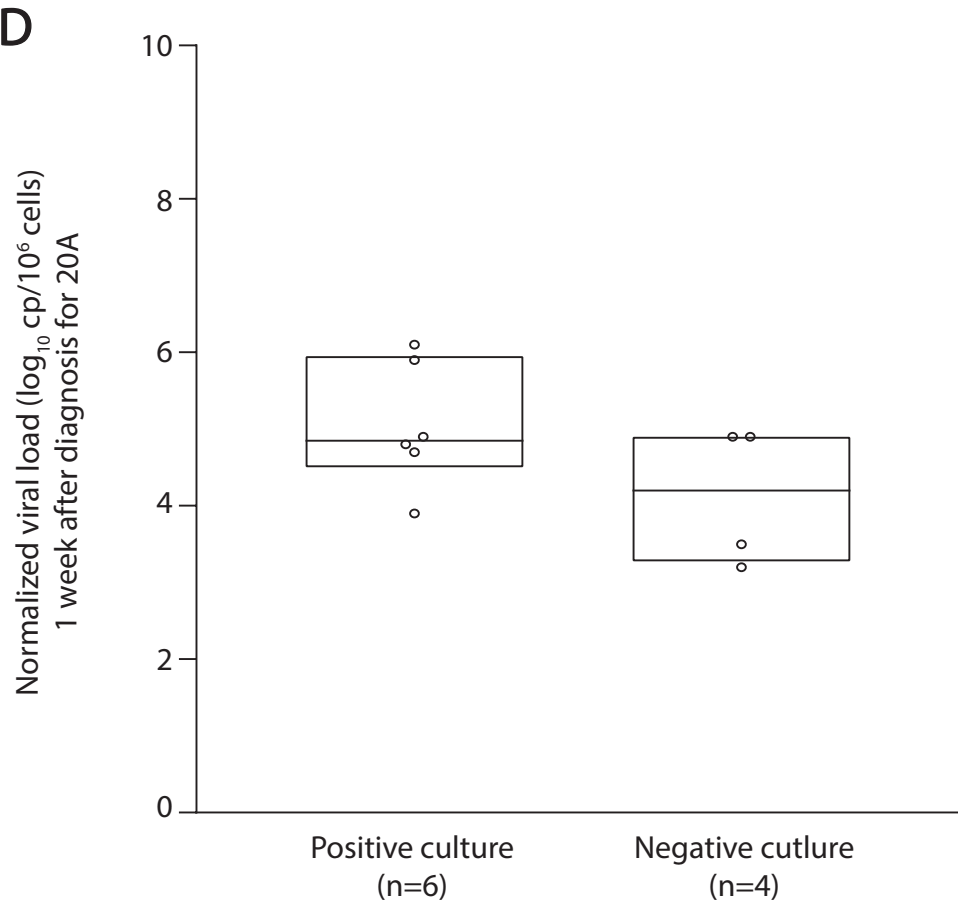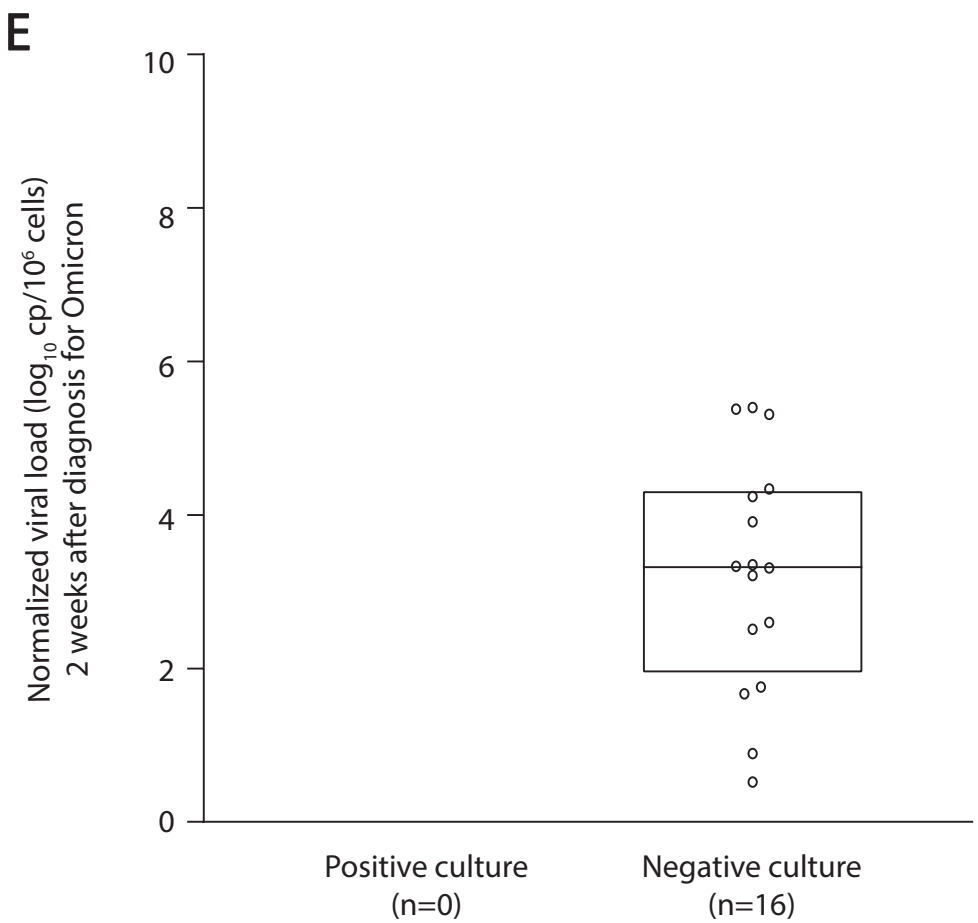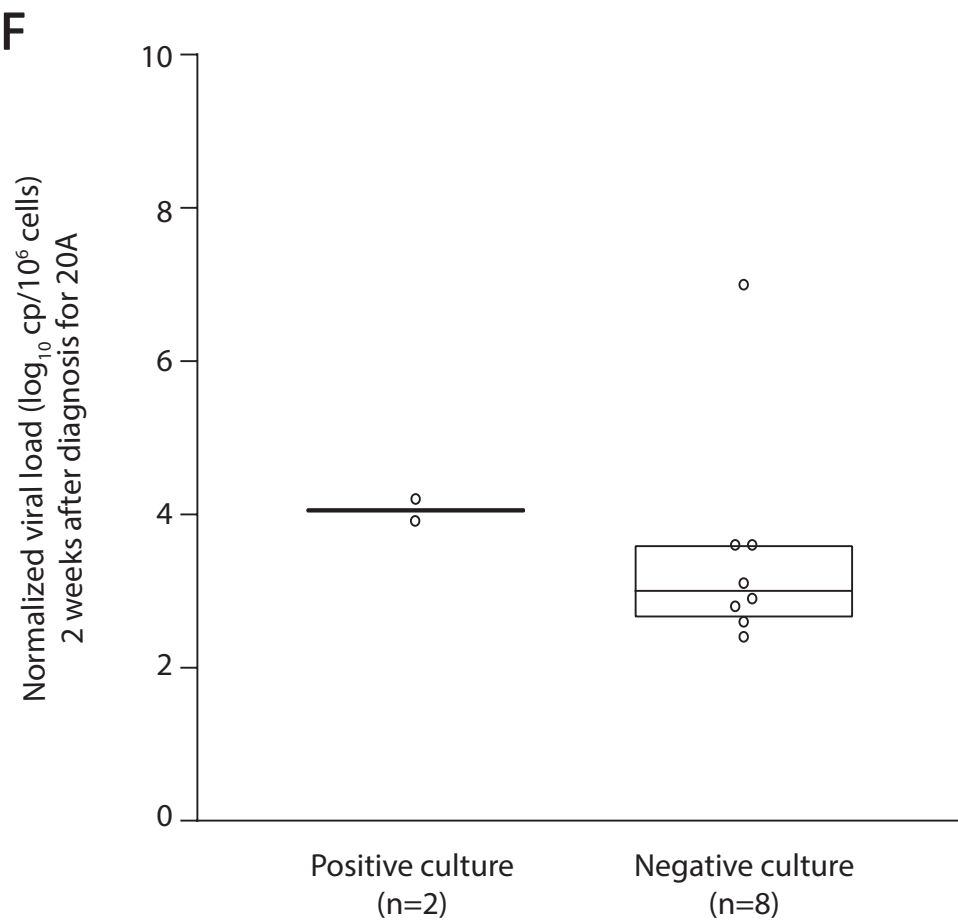

Supplement: Supplemental Material [file TEMI_A_2122578_SM5624.zip › Supplementary_material/Figure_S1_EMI_225703664.pdf]

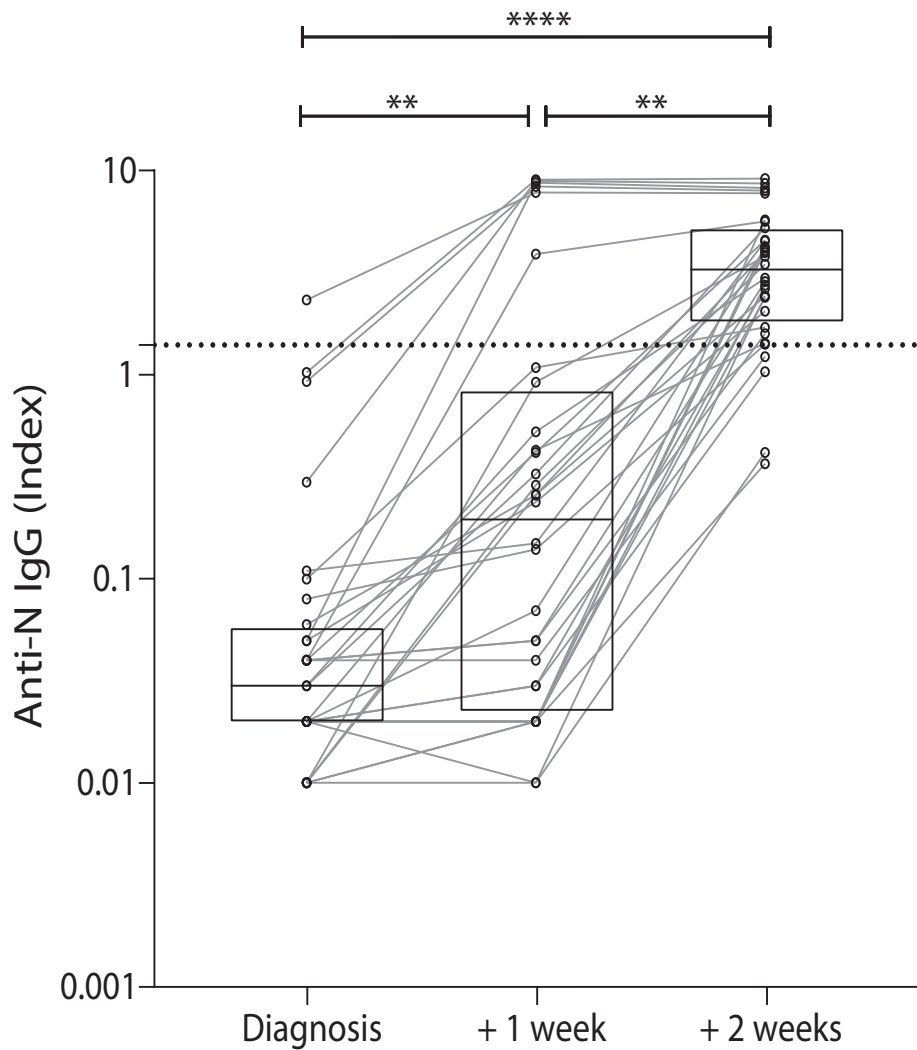

Supplement: Supplemental Material [file TEMI_A_2122578_SM5624.zip › Supplementary_material/Figure_S2_EMI_225703664.pdf]

**A**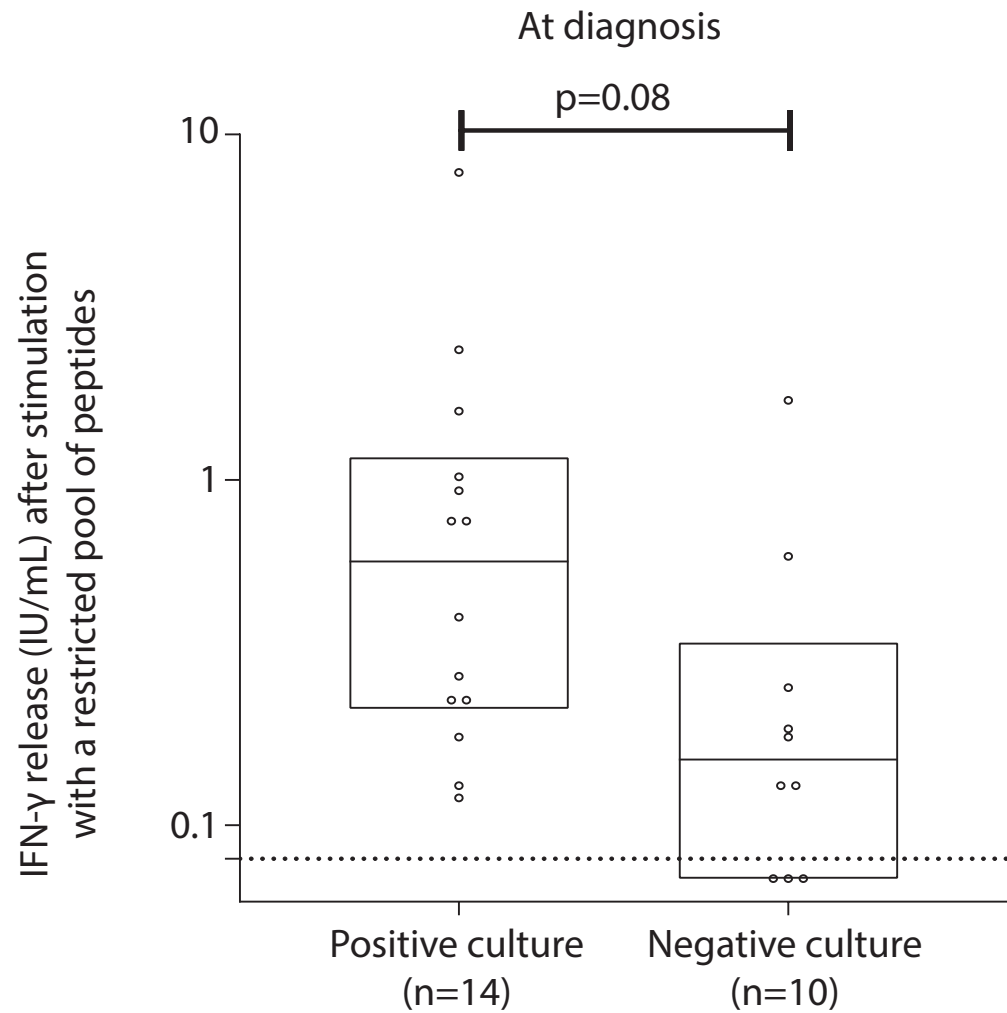**B**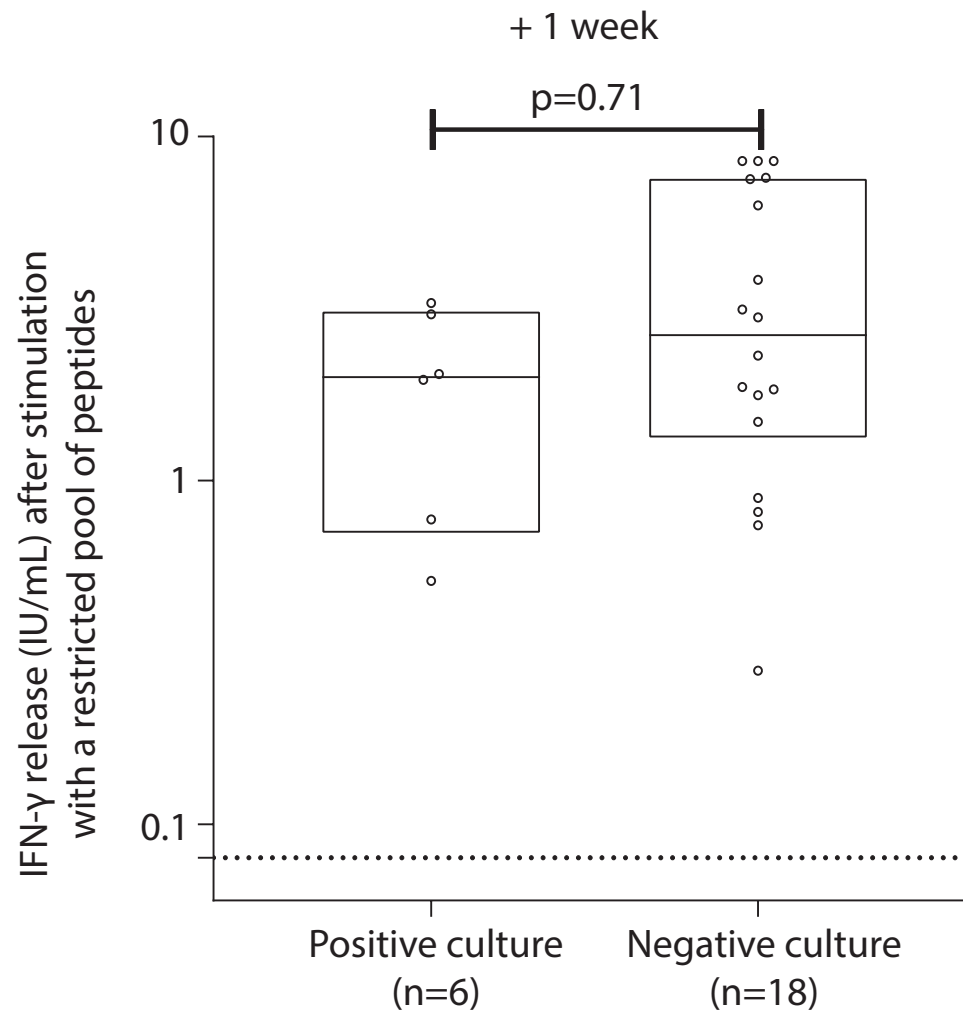

Supplement: Supplemental Material [file TEMI_A_2122578_SM5624.zip › Supplementary_material/Figure_S3_EMI_225703664.pdf]
